# Determinants of treatment pathway in renal colic: a size-stratified analysis from the emergency department of a tertiary center

**DOI:** 10.1007/s00240-026-01950-1

**Published:** 2026-02-12

**Authors:** Iulia Blajan, Benedikt Ebner, Yannic Volz, Matthias Klein, Christian G. Stief, Maria Apfelbeck, Michael Chaloupka

**Affiliations:** 1 Department of Urology, LMU Klinikum Ludwig- Maximilians-University Munich, Marchioninistr. 15, Munich, 81377 Germany; 2 Department of Neurology, LMU Klinikum, Munich, Germany; 3 Emergency Department, LMU Klinikum, Munich, Germany

**Keywords:** Urolithiasis, Renal colic, Emergency department, Computed tomography, Risk stratification

## Abstract

Acute flank pain due to ureteral calculi is a common presentation in emergency departments (EDs). Decisions between conservative management and inpatient intervention remain difficult in the absence of absolute indications. This study aims to identify size-stratified predictors to assist ED decision-making in ureterolithiasis. This was a single-center retrospective study of patients presenting in the ED of a tertiary center with acute renal colic between October 2020 and November 2024. A total of 752 patients with CT-confirmed ureteral stones were included and analyzed. Receiver operating characteristic (ROC) analysis identified a 5.65-mm threshold; operationalized as 6 mm in the subsequent analyses. After size-based stratification, uni- and multivariate regression analyses were conducted to identify predictors for inpatient admission in the <6 mm subgroup and predictors for discharge in the ≥6 mm subgroup, respectively. Overall, 77% of 1,194 patients were discharged and managed conservatively, and 23% underwent inpatient intervention. In the < 6 mm subgroup (*n* = 514; 20% intervened), multivariable analysis identified larger stone diameter (OR 1.65 per mm, 95% CI 1.09–2.59; *p* = 0.021, lower eGFR (OR 0.95 per mL/min, 95% CI 0.91–0.98; *p* = 0.002) and younger age (OR 0.96 per year, 95% CI [0.92, 0.99]; *p* = 0.024) as independent predictors for intervention. In the ≥ 6 mm subgroup (*n* = 238; 45% discharged), a distal location was the only independent predictor of discharge (OR 5.33, 95% CI 1.12–31.3; *p* = 0.045). A pragmatic 6-mm threshold, complemented by basic presentation markers, refines ED management beyond size alone. For stones < 6 mm, greater diameter and reduced renal function independently signaled the need for inpatient intervention, whereas age was inversely associated. For stones ≥ 6 mm, predominantly distal location favored discharge.

**Supplementary Information:**

The online version contains supplementary material available at 10.1007/s00240-026-01950-1.

## Introduction

Ureteral calculi are among the most frequent causes of acute flank pain and account for about 1% of emergency department (ED) visits [1, 2]. Management spans from analgesia with non-steroidal anti-inflammatory drugs (NSAIDs) and medical expulsive therapy (MET) with outpatient follow-up to urgent urinary diversion or primary ureteroscopy in selected cases [3, 4].

Despite the frequent clinical presentation with excruciating pain, studies show that the majority of ureteral and renal stones pass spontaneously [5].

Within this context, medical expulsive therapy (MET)- most commonly α-blockers- is frequently initiated in the emergency department and aims to facilitate the passage of the stone. Meta-analytic evidence suggests a modest, size- and location-dependent benefit (most apparent for distal stones>5 mm), with heterogeneous effects across trials [6–8].

In the presence of red flags (such as suspected or confirmed urosepsis, obstructive pyelonephritis or pyonephrosis, anuria, acute kidney injury, or rising creatinine due to obstruction) an emergency intervention is warranted. However, in the absence of these red flags, the decision between discharge and inpatient admission remains challenging. Stone size and location are established correlates of spontaneous passage, with larger and more proximally located stones showing lower passage rates [5]. Yet, size thresholds used to guide ED decision-making vary across studies, and size alone insufficiently captures the clinical complexity of obstructing stones [9]. Furthermore, despite evidence identifying which stones are likely to pass spontaneously, there is little evidence on the circumstances in which stones expected to pass are nevertheless operated on, and conversely, when stones that would usually require surgery are managed conservatively.

Accordingly, the objective of this study was to identify size-stratified predictors to support ED decision-making in patients with renal colic due to ureterolithiasis. Specifically, the focus lay on predictors associated with inpatient admission and subsequent surgical intervention versus conservative treatment. Within each stratum, we assessed clinical, laboratory, and computed-tomography variables as candidate predictors using univariable and multivariable logistic regression tailored to the respective outcome.

## Materials and methods

### Study design and patient cohort

This retrospective analysis was performed at the Department of Urology, LMU University Hospital, Munich, Germany. All patients presenting with acute renal colic due to urolithiasis at the interdisciplinary emergency department between October 2020 and November 2024 were screened for eligibility. In total, 1,194 patients were retrospectively identified. Diagnoses were retrieved using the International Classification of Diseases, 10th Revision, German Modification (ICD-10-GM), and the following codes were applied as inclusion criteria: N20.0 (renal calculus, nephrolithiasis, staghorn calculus), N20.1 (ureteral calculus), N20.2 (combined renal and ureteral calculus), and N20.9 (unspecified urinary calculus, including pyelonephritis associated with urinary calculi). Of these, 752 patients had radiographically confirmed ureteral calculi and were included in the final analysis, while patients with isolated renal calculi or with intravesical stones on CT indicating a spontaneously passed stone were excluded.

### Clinicopathologic and radiographic features

For all included patients, data were retrospectively extracted from the electronic medical records of the ED. Demographic variables comprised age, sex, and body mass index. Clinical history included the type, localization, duration, and intensity of pain as well as associated symptoms. Information on comorbidities, known anatomical anomalies, and chronic medication use was obtained to assess risk factors. Findings at presentation in the emergency department were documented, including results from physical examination, vital signs, ultrasound, urine analysis, laboratory parameters (such as creatinine, estimated glomerular filtration rate [eGFR], C-reactive protein, leucocyte count, and hemoglobin). Imaging data were obtained from computed tomography performed in the emergency department and included the number, anatomical location, density, and maximum diameter of ureteral stones. Therapeutic interventions at the time of presentation, particularly the administration of analgesics and antibiotic treatment, were systematically documented. The subsequent clinical course was also recorded, with outcomes categorized as discharge from the emergency department under MET, discharge following inpatient admission with conservative management (analgesia and MET), or admission requiring inpatient intervention. To clarify, inpatient intervention was defined as any urological operative procedure performed during the index hospitalization, including ureteral stent (double-J) placement, percutaneous nephrostomy, or primary ureteroscopy with stone extraction or fragmentation.

### Statistical analysis

Descriptive statistics were used to summarize patient characteristics and to compare patients discharged either directly from the emergency department or after inpatient admission without intervention to those who required inpatient admission and a surgical intervention. Continuous variables were reported as medians with interquartile ranges (IQRs), and categorical variables as absolute frequencies with corresponding percentages. For group comparisons, the Wilcoxon rank-sum test was applied to continuous variables, as the data did not meet the assumptions of normality required for parametric testing. Categorical variables were compared using Pearson’s chi-squared test when all expected cell counts were ≥ 5; in cases where this assumption was not fulfilled, Fisher’s exact test was employed. A two-sided p-value < 0.05 was considered statistically significant.

To determine the optimal cutoff value for stone size and subsequent treatment pathway, receiver operating characteristic (ROC) curve analysis was performed. Based on the identified threshold of 5.6 mm, patients were stratified into two subgroups (< 6 mm and ≥ 6 mm). In patients with stones < 6 mm, the outcome of interest was inpatient admission with intervention, while in patients with stones ≥ 6 mm, the outcome was discharge from the emergency department. Univariate and multivariate logistic regression analyses were subsequently conducted within each subgroup to identify independent predictors of these outcomes.

Missingness primarily reflected incomplete routine clinical documentation and laboratory or urine tests not obtained during ED care, rather than data loss during study processing. We quantified missingness at the variable level and report non-missing counts (N) for each variable in the baseline characteristics tables; additionally, variable-level missingness for all predictors used in regression analyses is summarized in Supplementary Table S1. The ROC analysis was performed using complete-case data, including only patients with non-missing values for the outcome and the predictor (stone size). Univariable logistic regression analyses were conducted using variable-specific complete-case analysis. Multivariable logistic regression models were conducted as complete-case analyses (listwise deletion), excluding patients with missing data in any included covariate; the effective sample size comprised of *N* = 99/238 for the ≥ 6 mm subgroup and *N* = 224/514 for the < 6 mm subgroup.

All statistical analyses were performed using R software (version 4.5.0, R Foundation for Statistical Computing, Vienna, Austria).

Written informed consent from participants was not required for anonymized retrospective analysis following the local guidelines of the Ethics Committee of the Ludwig-Maximilians University Munich. Ethical approval was obtained for the retrospective retrieval, analysis, and anonymized processing of existing patient data (project number: 25–0413; date of approval: 23 July 2025). The study was conducted in accordance with the Declaration of Helsinki.

## Results

### Baseline characteristics

Baseline characteristics are displayed in Table 1. Between October 2020 and November 2024, 1194 patients with acute renal colic due to urolithiasis presented to the interdisciplinary emergency department. Of these, 922/1194 (77%) were discharged (including those discharged after brief inpatient observation without intervention), whereas 272 (23%) were admitted for inpatient intervention. Among the 272 admitted patients, the majority underwent retrograde ureteral double-J stent placement (*n* = 240, 88.2%), while primary ureteroscopy was performed in 31 patients (11.4%), and antegrade double-J stent placement in one patient (0.4%). Patients requiring intervention were older (median 51.0, Interquartile Range (IQR) [39.0–61.5] vs. 45.0 [34.0–56.0] years; *p* < 0.001), while sex distribution and BMI were comparable between groups. Ambulance arrival was more common among patients who subsequently underwent intervention compared to those who did not present to the ED by ambulance (56% vs. 47%; *p* = 0.048).

Laboratory findings indicated greater systemic inflammation and renal impairment among patients undergoing intervention compared to discharged patients: creatinine was higher (1.1 [1.0–1.4] vs. 1.0 [0.9–1.2] mg/dL; *p* < 0.001), eGFR lower (70.0 [52.0–91.5] vs. 86.0 [70.0–102.0] mL/min; *p* < 0.001), CRP elevated (0.4 [0.2–2.0] vs. 0.2 [0.1–0.5] mg/dL; *p* < 0.001), and leukocyte count higher (10.7 [8.2–13.3] vs. 9.3 [7.5–11.6] G/L; *p* < 0.001). Urinary tract infections with nitrite positivity (8.4% vs. 3.3%; *p* < 0.001) and leukocyturia (48% vs. 39%; *p* = 0.012) were more frequent in the intervention group, whereas microhematuria and bacteriuria did not differ significantly. Urine cultures were tested more often positively in the intervention cohort compared to the conservatively treated cohort (14% vs. 5.7%; *p* = 0.008).

Imaging characteristics showed a clear gradient of stone burden and obstruction among patients requiring intervention compared to discharge from ED: proximal ureteral location was more common in 93/237 (39%) vs. 112/521 (21%) patients, while distal location was less common in 102/237 (43%) vs. 344/521 (66%) patients (overall *p* < 0.001), stone size was larger (6.4 [4.7–8.6] vs. 4.4 [3.4–5.6] mm; *p* < 0.001), and median Hounsfield units were higher (728.8 [475.1–962.1] vs. 472.6 [341.7–681.0]; *p* < 0.001) compared to patients not requiring intervention. Hydronephrosis was present in 91% of patients undergoing intervention compared with 67% of those discharged (*p* < 0.001). The duration of emergency department management was similar between groups (both 4.0 h; *p* = 0.9).

**Table 1:** Baseline characteristics of patients presenting to the emergency department (ED) with renal colic due to urolithiasis and their outcomes. Continuous variables are presented as median with interquartile range (IQR), categorical variables as proportions. N indicates the number of patients with available data for the respective variable. Bold values indicate statistical significance at *p* < 0.05. Abbreviations: BMI: body mass index. HU: Hounsfield units. IQR: interquartile range. ¹Median (Q1, Q3); n (%). ²Wilcoxon rank-sum test; pearson’s chi-squared test; fisher’s exact test

Characteristics	*N*	Discharge *N* = 922^1^	Inpatient admission and intervention *N* = 272^1^	*p*-value^2^
Age [years]	1,194	45.0 (34.0, 56.0)	51.0 (39.0, 61.5)	**< 0.001**
*Sex*	1,192			0.4
Female		250 (27%)	81 (30%)	
Male		670 (73%)	191 (70%)	
BMI [kg/m^2^]	426	26.6 (23.7, 30.0)	26.3 (23.7, 30.0)	0.7
*Arrival at the ED*	770			**0.048**
By ambulance		278 (47%)	102 (56%)	
Independently		309 (53%)	81 (44%)	
Diabetes mellitus	1,078	59 (7.2%)	24 (9.1%)	0.3
Hyperparathyroidism	1,080	0 (0%)	3 (1.1%)	**0.014**
*GI Diseases*	1,079			**0.019**
Crohn’s disease		19 (2.3%)	11 (4.2%)	
Ulcerative colitis		1 (0.1%)	3 (1.1%)	
*Pain character*	903			**0.019**
Colicky		564 (80%)	140 (72%)	
Constant		144 (20%)	55 (28%)	
Pain duration [h]	868	7.0 (3.0, 24.0)	10.0 (4.0, 48.0)	**< 0.001**
Nausea	570	217 (49%)	79 (61%)	**0.022**
Vomiting	572	147 (33%)	60 (48%)	**0.002**
Fever	816	15 (2.3%)	13 (7.6%)	**< 0.001**
Macrohematuria	575	65 (14%)	13 (11%)	0.3
Analgesia before ED presentation	1,194	859 (93%)	256 (94%)	0.6
History of stones	658	277 (54%)	89 (62%)	0.072
Systolic blood pressure on admission [mmHg]	849	145 (135, 160)	146 (133, 162)	> 0.9
Diastolic blood pressure on admission [mmHg]	848	88 (80, 97)	87 (78, 98)	0.8
Heart rate on admission [bpm]	828	75 (67, 86)	75 (67, 85)	0.5
Temperature on admission [°C]	827	36.6 (36.3, 36.9)	36.7 (36.3, 37.1)	0.02
Respiratory rate on admission	394	14 (14, 16)	14.5 (14, 16)	0.059
Creatinine [mg/dl]	1,181	1.0 (0.9, 1.2)	1.1 (1.0, 1.4)	**< 0.001**
eGFR [ml/min]	1,174	86.0 (70.0, 102.0)	70.0 (52.0, 91.5)	**< 0.001**
CRP [mg/dl]	1,020	0.2 (0.1, 0.5)	0.4 (0.2, 2.0)	**< 0.001**
Leukocytes [G/l]	1,179	9.3 (7.5, 11.6)	10.7 (8.2, 13.3)	**< 0.001**
Urine: Nitrite positive	1,073	27 (3.3%)	21 (8.4%)	**< 0.001**
Urine: Leukocyturia	1,073	323 (39%)	120 (48%)	**0.012**
Urine: Microhematuria	1,074	578 (70%)	179 (72%)	0.6
Urine: Bacteriuria	500	304 (80%)	104 (86%)	0.2
Urine culture positive	325	11 (5.7%)	19 (14%)	**0.008**
*Location of symptomatic stone*	758			**< 0.001**
Proximal		112 (21%)	93 (39%)	
Mid ureter		65 (12%)	42 (18%)	
Distal		344 (66%)	102 (43%)	
Largest ureteral stone diameter [mm]	752	4.4 (3.4, 5.6)	6.4 (4.7, 8.6)	**< 0.001**
Mean HU	849	472.6 (341.7, 681.0)	728.8 (475.1, 962.1)	**< 0.001**
Hydronephrosis	1,194	619 (67%)	248 (91%)	**< 0.001**
ED treatment duration [h]	1,179	4.0 (3.0, 5.0)	4.0 (3.0, 6.0)	0.9

### ROC analysis and selection of a ureteral stone size cut-off for stratification

**Fig. 1:**
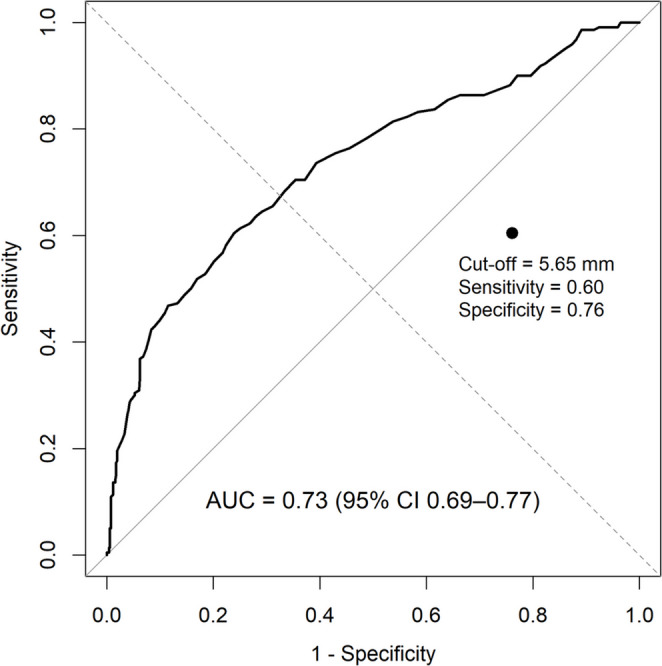
Receiver operating characteristics (ROC) analysis for stone size predicting urological intervention Abbreviations: AUC: Area under curve

In the overall cohort, receiver operating characteristic (ROC) analysis of stone size to discriminate inpatient surgical intervention versus discharge identified an optimal threshold of 5.65 mm (Youden index) with an AUC 0.74, indicating good discriminatory ability. This threshold yielded a sensitivity of 60% and a specificity of 76% (Fig. 1). For subsequent analyses, we operationalized this data-driven cut-off as 6 mm.

### Patients with ureteral stones < 6 mm: baseline characteristics and predictors for need for intervention

Within the < 6 mm subgroup, 414 of 514 (80%) patients were discharged. Of these, 65 patients (15%) revisited the ED due to the same stone. Regarding the stone location of discharged patients 73/506 (18%) presented with proximal, 50/506 (12%) presented with mid-ureteral and 283/506 (70%) exhibited distal calculi. 100/514 (20%) required inpatient surgical intervention. Among these, proximal stones accounted for 20/100 (20%) cases, 15/100 (15%) stones were located in the mid ureter and 65/100 (65%) were located in the distal ureter. The intervention group reported a markedly longer symptom duration (11.0 [5.0–72.0] vs. 5.0 [3.0–20.0] hours; *p* < 0.001). Laboratory markers indicated greater inflammatory burden and renal impairment among those requiring intervention, with higher creatinine (1.2 [1.0–1.4] vs. 1.0 [0.9–1.2] mg/dL; *p* < 0.001), lower eGFR (71.5 [56.0–95.0] vs. 84.0 [69.0–100.0] mL/min; *p* < 0.001), higher CRP (0.3 [0.1–1.4] vs. 0.2 [0.1–0.4] mg/dL; *p* < 0.001), and higher leukocyte counts (10.9 [7.8–13.6] vs. 9.5 [7.6–11.9] G/L; *p* = 0.012). Urinary tract infections with Nitrite positivity were more frequent in the intervention group (5.0% vs. 2.0%; *p* = 0.032), whereas microhematuria was less frequent (69% vs. 81%; *p* = 0.011) Stone characteristics still differed within this size band: the maximum ureteral stone diameter was larger in patients undergoing intervention (4.5 [3.3–5.1] vs. 4.0 [3.1–4.7] mm; *p* = 0.002). The characteristics of patients in the <6 mm subgroup are presented in Table 2.

Regarding the univariate analysis (Table 3), higher inflammatory and renal impairment markers were associated with increased odds of intervention: CRP (OR 1.27 per mg/dL, 95% CI 1.15–1.44; *p* < 0.001) and creatinine (OR 2.09 per mg/dL, 95% CI 1.28–3.82; *p* = 0.011) were positively associated, whereas eGFR was inversely associated (OR 0.98 per mL/min, 95% CI 0.97–0.99; *p* < 0.001). A larger ureteral stone diameter, even within this small-size band, also predicted intervention (OR 1.38 per mm, 95% CI 1.13–1.70; *p* = 0.002).

In the adjusted model (Table 3), greater stone diameter (OR 1.65 per mm, 95% CI 1.09–2.59; *p* = 0.021) and lower eGFR (OR 0.95 per mL/min, 95% CI 0.91–0.98; *p* = 0.002) independently predicted intervention, while age was inversely associated (OR 0.96 per year, 95% CI 0.92–0.99; *p* = 0.024). The inflammatory markers did not retain significance.

### Patients with ureteral stones ≥ 6 mm: baseline characteristics and predictors for discharge

Among 238 patients with stones ≥ 6 mm, 107 (45%) were discharged. Of these, 20 patients (19%) returned to the ED due to the same stone and 131 (55%) were admitted for intervention. The distribution of stone locations differed between groups. While 38/105 (36%) of the discharged patients presented with proximal stones, 14/105 (13%) with mid-ureteral stones, and 53/105 (50%) with distal stones, the majority of the admitted patients, 72/131 (55%), exhibited a proximal stone location, followed by 34/131 (26%) with distal stones, and a minority with mid-ureteral stones (25/131, 19%). Discharged patients tended to be younger (49.0 [41.0–57.0] vs. 53.0 [41.0–65.0] years; *p* = 0.07), with better renal indices: lower creatinine (1.0 [0.9–1.2] vs. 1.1 [1.0–1.4] mg/dL; *p* = 0.002), higher eGFR (81.0 [68.0–94.0] vs. 69.0 [49.0–87.0] mL/min; *p* < 0.001) and, on imaging, were more likely to present with distal stones (50% vs. 26%; *p* < 0.001) and less hydronephrosis (85% vs. 95%; *p* = 0.013). Vomiting was also less common in the discharged group (*p* = 0.01). Baseline characteristics of patients with ureteral calculi ≥ 6 mm are displayed in Table 2.

Univariate analysis showed higher eGFR, smaller diameter, distal location and absence of hydronephrosis favoring discharge, whereas higher creatinine and vomiting reduced it (all *p* ≤ 0.016) (Table 4).

In the multivariate analysis (Table 4), distal stone location (vs. proximal) was the strongest independent predictor of hospital discharge (OR 5.33, 95% CI 1.12–31.3; *p* = 0.045).

**Table 2:** Baseline characteristics of patients presenting to the emergency department (ED) with renal colic and CT-confirmed ureteral stones, stratified by stone size and management. Continuous variables are presented as median with interquartile range (IQR), categorical variables as n (%). N indicates the number of patients with available data for the respective variable. Bold values indicate statistical significance at *p* < 0.05. Abbreviations: BMI: body mass index. HU: Hounsfield units. IQR: interquartile range. ^1^Median (Q1, Q3); n (%). ^2^Wilcoxon rank sum test; pearson’s Chi-squared test; fisher’s exact test

Characteristics	*N*	Maximum ureteral stone size <6 mm			*N*	Maximum ureteral stone size ≥6 mm		
		Discharge *N* = 414^1^	Inpatient admission and intervention *N* = 100^1^	*p*-value^2^		Discharge *N* = 107^1^	Inpatient admission and intervention *N* = 131^1^	*p*-value^2^
Age [years]	514	44.5 (33.0, 56.0)	49.0 (37.0, 60.0)	0.066	238	49.0 (41.0, 57.0)	53.0 (41.0, 65.0)	0.07
BMI [kg/m^2^]	164	26.3 (23.2, 29.6)	26.7 (23.5, 30.8)	0.8	164	26.9 (24.4, 31.4)	26.3 (23.7, 30.1)	0.4
*Sex*	513			0.7	238			0.15
Female		103 (25%)	23 (23%)			23 (21%)	39 (30%)	
Male		310 (75%)	77 (77%)			84 (79%)	92 (70%)	
*Arrival at the ED*	347			> 0.9	158			0.1
By ambulance		142 (52%)	40 (53%)			30 (42%)	47 (55%)	
Independently		129 (48%)	36 (47%)			42 (58%)	39 (45%)	
Diabetes mellitus	462	27 (7.4%)	6 (6.3%)	0.7	221	8 (8.5%)	15 (12%)	0.4
*Pain character*	412			0.1	175			**0.018**
Colicky		283 (84%)	59 (77%)			67 (83%)	63 (67%)	
Constant		52 (16%)	18 (23%)			14 (17%)	31 (33%)	
Pain duration [h]	386	5.0 (3.0, 20.0)	11.0 (5.0, 72.0)	**< 0.001**	168	11.0 (4.0, 48.0)	10.0 (4.0, 36.0)	0.7
Nausea	271	114 (52%)	34 (64%)	0.12	106	23 (55%)	40 (63%)	0.4
Vomiting	269	81 (37%)	23 (46%)	0.2	102	11 (27%)	32 (52%)	0.01
Fever	360	5 (1.7%)	3 (4.6%)	0.2	157	2 (2.7%)	6 (7.2%)	0.3
Macrohematuria	255	26 (12%)	2 (4.5%)	0.2	110	10 (21%)	8 (13%)	0.2
Analgesia before ED presentation	514	390 (94%)	94 (94%)	> 0.9	238	100 (93%)	122 (93%)	> 0.9
History of stones	278	104 (47%)	32 (57%)	0.2	126	35 (61%)	47 (68%)	0.4
Systolic blood pressure on admission [mmHg]	395	147.0 (137.0, 160.0)	147.0 (133.0, 165.0)	0.6	163	150.5 (136.0, 163.0)	149.0 (134.0, 165.0)	0.8
Diastolic blood pressure on admission [mmHg]	394	89.0 (81.0, 99.0)	89.0 (80.0, 99.0)	0.7	163	89.5 (80.0, 98.0)	88.0 (78.0, 98.0)	0.4
Heart rate on admission [bpm]	391	76.0 (67.0, 87.0)	75.0 (67.0, 83.0)	> 0.9	158	76.0 (65.0, 86.0)	76.0 (68.0, 86.0)	0.8
Temperature on admission [°C]	369	36.6 (36.3, 36.9)	36.7 (36.3, 37.0)	0.1	161	36.7 (36.4, 37.0)	36.6 (36.3, 37.0)	> 0.9
Respiratory rate on admission	184	14.0 (14.0, 16.0)	15.0 (14.0, 16.0)	0.079	79	14.0 (14.0, 16.0)	14.0 (14.0, 16.0)	0.9
Creatinine [mg/dl]	514	1.0 (0.9, 1.2)	1.2 (1.0, 1.4)	**< 0.001**	235	1.0 (0.9, 1.2)	1.1 (1.0, 1.4)	**0.002**
eGFR [ml/min]	508	84.0 (69.0, 100.0)	71.5 (56.0, 95.0)	**< 0.001**	235	81.0 (68.0, 94.0)	69.0 (49.0, 87.0)	**< 0.001**
CRP [mg/dl]	443	0.2 (0.1, 0.4)	0.3 (0.1, 1.4)	**< 0.001**	204	0.4 (0.1, 0.9)	0.3 (0.2, 1.8)	0.5
Leukocytes [G/l]	513	9.5 (7.6, 11.9)	10.9 (7.8, 13.6)	**0.012**	235	9.8 (7.9, 11.5)	10.6 (8.2, 12.6)	**0.05**
Urine: Nitrite positive	514	9 (2%)	5 (5.0%)	**0.032**	238	5 (5%)	11 (8%)	0.5
Urine: Leukocyturia	514	194 (47%)	38 (38%)	0.11	238	51 (48%)	74 (56%)	0.2
Urine: Microhematuria	514	334 (81%)	69 (69%)	**0.011**	238	90 (84%)	99 (76%)	0.11
Urine: Bacteriuria	514	386 (93%)	93 (93%)	> 0.9	238	98 (92%)	125 (95%)	0.2
Urine culture positive	146	3 (3.3%)	5 (9.3%)	0.15	87	2 (7.1%)	10 (17%)	0.3
*Location of symptomatic stone*	506			0.6	236			**< 0.001**
Proximal		73 (18%)	20 (20%)			38 (36%)	72 (55%)	
Mid ureter		50 (12%)	15 (15%)			14 (13%)	25 (19%)	
Distal		283 (70%)	65 (65%)			53 (50%)	34 (26%)	
Largest ureteral stone diameter [mm]	514	4.0 (3.1, 4.7)	4.5 (3.3, 5.1)	**0.002**	238	6.8 (6.3, 8.1)	8.4 (7.1, 10.2)	**< 0.001**
Mean HU	497	434.7 (323.0, 570.0)	464.9 (341.8, 686.5)	0.067	238	753.5 (554.3, 882.1)	914.0 (681.4, 1,138.4)	**< 0.001**
Hydronephrosis	514	352 (85%)	91 (91%)	0.12	238	91 (85%)	124 (95%)	0.013
ED treatment duration [h]	509	4.0 (3.0, 6.0)	4.0 (3.0, 5.0)	0.2	234	4.0 (3.0, 5.0)	4.0 (3.0, 6.0)	0.1

**Table 3:** Uni- and multivariate analysis of the <6 mm ureteral stone subgroup with the outcome inpatient admission and intervention. Bold values indicate statistical significance at *p* < 0.05. Abbreviations: CI = Confidence Interval, OR = Odds ratio

Variable	Univariate analysis			Multivariate analysis		
	OR	95%CI	*p*-value	OR	95%	*p*-value
Age [years]	1.01	1.00, 1.03	0.061	0.96	0.92, 0.99	**0.024**
*Sex*						
Female	–	–		–	–	
Male	1.11	0.67, 1.90	0.7	0.53	0.19, 1.60	0.2
*Arrival at the ED*						
By ambulance	–	–				
Independently	0.99	0.59, 1.65	> 0.9			
Fever	2.81	0.56, 11.7	0.2	1.29	0.08, 12.5	0.8
Vomiting	1.45	0.78, 2.70	0.2	2.01	0.79, 5.22	0.14
CRP [mg/dl]	1.27	1.15, 1.44	**< 0.001**	1.21	1.03, 1.51	0.052
Leukocytes [G/l]	1.02	0.99, 1.07	0.2	1.16	1.00, 1.35	0.055
Creatinine [mg/dl]	2.09	1.28, 3.82	0.011	0.92		0.9
eGFR [ml/min]	0.98	0.97, 0.99	**< 0.001**	0.95	0.91, 0.98	**0.002**
Urine: Leukocyturia	0.7	0.44, 1.08	0.11	0.56	0.19, 1.49	0.3
*Location of symptomatic stone*						
Proximal	–	–		–	–	
Mid ureter	1.1	0.51, 2.33	0.8	0.65	0.11, 3.30	0.6
Distal	0.84	0.48, 1.50	0.5	1.11	0.34, 4.12	0.9
Hydronephrosis	1.78	0.89, 3.96	0.12	3.42	0.57, 67.0	0.3
Largest ureteral stone diameter [mm]	1.38	1.13, 1.70	**0.002**	1.65	1.09, 2.59	**0.021**

**Table 4:** Uni- and multivariate analysis of the ≥6 mm ureteral stone subgroup with the outcome discharge. Bold values indicate statistical significance at *p* < 0.05. Abbreviations: CI = Confidence Interval, OR = Odds ratio

Variable	Univariate analysis			Multivariate analysis		
	OR	95% CI	*p*-value	OR	95% CI	*p*-value
Age [years]	0.98	0.97, 1.00	0.071	0.99	0.91, 1.08	0.8
*Sex*						
Female	–	–		–	–	
Male	1.55	0.86, 2.83	0.15	11.8	1.22, 231	0.056
*Arrival at the ED*						
By ambulance	–	–		–	–	
Independently	1.69	0.90, 3.19	0.1	4.03	0.84, 24.4	0.1
Fever	0.36	0.05, 1.60	0.2			
Vomiting	0.33	0.14, 0.77	**0.011**	0.73	0.17, 3.03	0.7
CRP [mg/dl]	0.96	0.88, 1.04	0.3	1.07	0.64, 1.44	0.7
Leukocytes [G/l]	0.98	0.91, 1.03	0.4	0.82	0.63, 1.05	0.13
Creatinine [mg/dl]	0.21	0.08, 0.52	**0.001**	0.01	0.00, 43.7	0.3
eGFR [ml/min]	1.02	1.01, 1.03	**< 0.001**	0.97	0.84, 1.10	0.7
Urine: Leukocyturia	0.7	0.42, 1.17	0.2	1.77	0.39, 9.15	0.5
*Location of symptomatic stone*						
Proximal	–	–		–	–	
Mid ureter	1.06	0.49, 2.26	0.9	0.41	0.04, 2.91	0.4
Distal	2.95	1.66, 5.34	**< 0.001**	5.33	1.12, 31.3	**0.045**
Hydronephrosis	0.32	0.12, 0.79	**0.016**			
Largest ureteral stone diameter [mm]	0.74	0.63, 0.84	**< 0.001**	0.73	0.43, 1.14	0.2

## Discussion

The therapeutic decision-making for patients with renal colic remains challenging in many cases. Particularly when no absolute indications for intervention are present, either endoscopic intervention or conservative management may be appropriate at times, even when the stone size appears atypical for that strategy. In this single-center retrospective cohort of 1,194 patients presenting to the emergency department with renal colic, a ROC analysis identified a maximum diameter of 5.65 as the optimal size threshold for discriminating discharge versus inpatient intervention. For all subsequent analyses, this data-derived value was operationalized as 6 mm to enhance interpretability and reproducibility and to accommodate CT measurement imprecision and interobserver variability, while also aligning with commonly used clinical size bands. After size-based stratification, multivariate models showed that in the < 6 mm subgroup, larger maximal stone diameter (OR 1.65 per mm, 95% CI 1.09–2.59; *p* = 0.021) and lower eGFR (OR 0.95 per mL/min, 95% CI 0.91–0.98; *p* = 0.002) independently correlated with inpatient intervention, whereas older age (OR 0.96 per year, 95% CI 0.92–0.99; *p* = 0.024) was inversely associated. In the ≥ 6 mm subgroup, the adjusted analysis for inpatient intervention identified distal ureteral location (vs. proximal) as the only independent signal (OR 5.33, 95% CI 1.12–31.3; *p* = 0.045), albeit with wide confidence intervals indicating imprecision and potential collinearity in this group.

The ED decision management for ureterolithiasis involves balancing symptom management, risk of infection or obstruction, resource utilization, and patient preferences. Current guidelines endorse selective MET and emphasize shared decision-making, particularly for distal stones > 5 mm, where benefit is most plausible yet still modest [10, 11]. Extensive trials and population data further show that many patients are discharged from the ED and only a subset require subsequent procedures, underscoring the need for practical predictors at first contact to avoid both premature discharge and unnecessary admission [12, 13]. Consistent with these patterns, the majority of patients in the present cohort were managed conservatively and discharged (77%).

By integrating a clinically intuitive 6-mm cutoff with basic presentation data (eGFR/creatinine, CRP/leukocytes, hydronephrosis, stone location and size), our method reflects how ED teams typically triage patients. It also supports existing guidelines that emphasize size and location, while allowing for additional operational tools that consider factors such as renal and inflammatory status, as well as obstruction surrogates.

Thus, our findings have direct clinical implications and may be translated into a simple, two-step imaging-based decision algorithm for patients with CT-confirmed ureterolithiasis. First, patients presenting with a renal colic and clinical red-flags such as fever, suspected urosepsis, elevated infection markers, anuria or acute kidney injury markers should be prioritized for admission and urgent urinary diversion disregarding stone size. Second, among clinically stable patients, a size-stratified model centered around the 6 mm threshold could support shared decision-making. In stones < 6 mm, increasing stone diameter and reduced renal function independently signaled a higher likelihood of inpatient intervention, whereas in stones ≥ 6 mm, distal ureteral location emerged as the most consistent factor favoring discharge. This framework complements existing clinical prediction tools such as the STONE or the CHOKAI score, which were primarily designed to estimate the probability of ureterolithiasis at presentation and to guide diagnostic imaging decisions [14, 15]. Notably, the CHOKAI score incorporates clinical features including age, sex, pain duration, prior stone history, hematuria, and costovertebral angle tenderness, and has demonstrated improved diagnostic accuracy compared with the STONE score in certain populations [16, 17]. Significantly, CHOKAI score components also correlate with stone size and location, underscoring the relevance of anatomical burden for subsequent risk stratification and management planning. Beyond diagnostic scores, several nomograms and machine-learning models have been proposed to predict spontaneous stone passage (SSP) or the need for intervention. Large multicenter nomograms incorporate stone size, ureteral location, hydronephrosis or hydroureter, inflammatory markers, and the use of medical expulsive therapy to generate individualized SSP probabilities, thereby supporting shared decision-making regarding conservative versus early interventional management [18, 19]. Furthermore, machine-learning techniques have shown high predictive accuracy for SSP and surgical intervention, using factors such as stone size and location, duration of pain, repeat ED visits, and vital signs [20, 21]. While these models provide more precise risk assessments, their complexity and dependence on complete datasets may restrict immediate use in fast-paced ED settings.

In this context, the current findings support a complementary and easily applicable strategy that relies on routinely available CT and laboratory findings already embedded into ED workflows. Further research is necessary to externally validate this method and to evaluate its integration into a straightforward point-based risk score.

The present findings align with guideline emphasis on stone size and location as primary drivers of management. Multiple cohort and ED-based studies have identified stone size as a principal predictor of inpatient admission and urologic intervention, with risk rising sharply beyond the mark of 5–6 mm [22, 23]. Exemplarily, Samson et al. identified a 5 mm threshold as a solid predictor for surgical need. The study reported a mean size of 7 mm for patients requiring interventions, significantly larger than the conservatively managed cohort, with a mean size of 4.1 mm [24]. Further research, including a comprehensive multi-center ED-based study, identified early intervention benefiting larger stone sizes ≥ 7 mm and increasing morbidity for stones < 5 mm, supporting size-anchored decision making [25].

Moreover, the stone location also consistently emerges as a pivotal determinant influencing inpatient admission with subsequent intervention. Foundational and contemporary literature demonstrates that ureteral calculi located proximally or in the mid-ureter are associated with higher rates of intervention compared to distal stones, due to lower rates of spontaneous passage and increased risk of persistent pain, obstruction, and emergency department revisits [26–28]. In the present analysis, this gradient was also evident after size-based stratification. The distal stone location was independently associated with higher odds of discharge compared with the proximal location (OR 5.33, 95% CI 1.12–31.3; *p* = 0.045). These findings are consistent with prior reports of greater spontaneous passage and lower intervention rates for distal stones. Accordingly, a distal location may support conservative management, even among larger stones, when risk markers are favorable.

Contemporary EAU guidance recommends considering α-blockers for distal ureteral stones > 5 mm and underscores that disposition should integrate stone characteristics with clinical status-consistent with our use of a pragmatic 6-mm threshold and ED-available variables [11].

ED cohort studies likewise report that renal impairment, infection markers and greater stone burden increase the likelihood of admission and subsequent intervention. Previous reports found that larger stones, kidney injury or infection, need for opioids, and hydronephrosis were associated with admission, and that predictors of intervention differ by initial disposition, paralleling our observation that, in < 6 mm stones, lower eGFR and larger diameter independently tracked with intervention, while in ≥ 6 mm stones, features such as distal location and smaller diameter within the band supported discharge [23].

Interestingly, the present multivariate analysis revealed a modest inverse association between older age and inpatient admission followed by urological intervention in the < 6 mm subgroup. This may be the effect of clinical selection and residual confounding. Older patients often have more comorbidities, higher anaesthetic risks, or treatment with anticoagulant agents, all of which may influence clinicians to pursue outpatient management rather than admission, especially if their conditions are stable or hospitalization risks outweigh benefits. Additionally, senior patients tend to display atypical or less severe symptoms, possibly causing healthcare providers to underestimate the need for admission or favor outpatient treatment [29]. Although our adjusted model considered factors like renal function, inflammatory markers, stone size, location, and hydronephrosis, residual and unmeasured confounding factors remain possible. Therefore, the age effect should be regarded as a practical indicator of clinical decision-making rather than a biological determinant.

In patients with stones ≥ 6 mm, distal ureteral location emerged as the only independent factor associated with discharge. At the same time, renal function parameters that were significant in univariate analyses (creatinine OR 0.21, 95% CI 0.08, 0.52, *p* = 0.001 and eGFR OR 1.02, 95% CI1.01, 1.03, *p* < 0.001) lost significance after adjustment. This pattern suggests that impaired renal function may represent a downstream consequence of stone-related obstruction rather than an independent determinant of discharge decisions. Proximal stones of this size are more likely to cause sustained obstruction and functional impairment [30]. In contrast, distal stones are associated with a higher likelihood of spontaneous passage, favoring outpatient management. After accounting for stone location, renal function appears to have a limited impact on the decision-making process, indicating a potential mediating role of ureteral location. In addition, stone size, location, and renal function are closely interrelated, and collinearity among these variables may have attenuated individual effects in the multivariable model. The wide confidence intervals observed for distal location further suggest limited precision and reduced statistical power within this stratified subgroup, underscoring the need for cautious interpretation of adjusted estimates.

Importantly, the identified predictors should be interpreted as determinants of emergency department disposition and treatment decisions rather than as predictors of the natural history of ureteral stone passage. Since disposition decisions were made with full awareness of CT findings, the observed associations reflect clinical risk assessment and perceived need for intervention at the point of care. This distinction is particularly relevant in the ED setting, where management prioritizes anticipated risk and feasibility of outpatient care over spontaneous passage probability alone.

Imaging literature increasingly explores CT markers beyond size and location. Studies highlight Hounsfield units, hydronephrosis, and emerging parameters such as ureteral wall thickness as correlates of spontaneous passage or obstruction risk. Our inclusion of HU and hydronephrosis, and the observed association between distal location and discharge in the ≥ 6 mm group, are consistent with these reports and reinforce the relevance of simple CT surrogates at the point of care [31, 32].

In the ≥ 6 mm analysis, fever, bacteriuria, and hydronephrosis were excluded due to perfect separation with the modelled endpoint (OR = 0). This pattern aligns with the near-absence of discharges among patients exhibiting these features. Apart from laboratory and imaging parameters, our study’s findings also underscore the significant role of the patient’s overall clinical impression. Impairments that could, in principle, be adequately managed conservatively were observed significantly more often in the group of patients who underwent urinary diversion compared to conservatively managed patients. Patients who received an intervention were significantly more likely to present to the ED vehicle-bound (e.g., via ambulance), had a longer duration of pain, and more frequently experienced nausea and vomiting compared to patients managed conservatively. The influence of pain duration and intensity on the likelihood of urinary diversion has also been demonstrated in prior single-center studies with small cohorts [23]. However, an effect of nausea and vomiting has not been established to date.

Some limitations of this study must be acknowledged. First, its single-center, retrospective design introduces potential selection and information bias, accounting for limited generalizability. All patients were treated at a tertiary-care center with continuous urological availability, standardized emergency department pathways, and unrestricted access to computed tomography. Center-specific practice patterns, including timing of urological consultation and availability of endoscopic intervention, may differ from those in community hospitals or resource-limited settings. As a result, disposition decisions observed in this cohort may reflect center-specific workflows and resource availability rather than universally applicable criteria. In addition, referral patterns may have influenced case mix. As a tertiary center, this institution may receive a higher proportion of complex or recurrent stone cases, prior urological history, or interfacility transfers, while less severe cases may have been managed entirely in outpatient settings and not captured. Together, these factors may limit the external validity of the derived 6-mm threshold and size-stratified predictors, underscoring the need for external validation in multicenter cohorts and across different health-system contexts.

Case inclusion was restricted to presentations routed through the interdisciplinary emergency department. During daytime hours (approximately 08:00–15:00), urgent urological cases may be triaged via the outpatient clinic rather than the ED, potentially creating a route-of-care and time-of-presentation bias. Clinical documentation was occasionally sparse, resulting in missing data for history and symptom variables and reducing the effective sample size for subgroup and multivariable analyses. Variable-level missingness for the predictors used in the regression analyses is provided in the Supplementary Table 1. As a consequence, subgroup analyses and especially multivariable models were based on reduced effective sample sizes, and events per variable were limited in some models. This increases imprecision and widens confidence intervals, and it can elevate the risk of sparse-data bias, separation, and collinearity.

Second, although computed tomography protocols were standardized, interobserver variation and rounding to whole millimeters introduce measurement error, particularly near the size threshold. Hydronephrosis was mainly recorded as a binary variable rather than by graded severity, and stone location was categorized coarsely (proximal/mid/distal). In most cases, both ultrasound and CT were performed, and clinicians made decisions with full awareness of CT findings. Given the established link between stone size and spontaneous passage, it cannot be determined whether patients with larger stones consistently received an adequate trial of conservative management before admission or intervention. Accordingly, management informed by prognostic imaging may have introduced confounding by indication, meaning that the identified predictors primarily reflect clinical decision-making rather than the underlying natural history of stone passage.

Third, provider- and system-level factors likely influenced care pathways but were not fully captured. Management varied among clinicians, particularly in terms of antibiotic usage, administered analgesia, and disposition decisions. Moreover, this dataset did not include determinants such as insurance status, race, ethnicity, and weekday at presentation, all factors which were previously linked to variation in inpatient procedures, were not available in our dataset [33, 34]. As a result, generalizability to other regions and health-system contexts may be limited.

Finally, outcome assessment was confined to initial post-ED disposition and in-hospital intervention; longitudinal endpoints were not systematically recorded. Among discharged patients, it was known whether individuals re-presented for the same stone (*n* = 65 [15%] in the < 6-mm subgroup; *n* = 20 [19%] in the ≥ 6-mm subgroup), but the number of subsequent visits and outcome (stone passage or interventions) were not recorded. Patients may also have sought care at other hospitals or clinics, resulting in incomplete follow-up and potential misclassification of outcomes.

Overall, this report proves that using a pragmatic 6-mm threshold, ED decision-making in renal colic can be guided by a readily available set of markers, rather than size alone. For stones < 6 mm, greater diameter and reduced renal function independently signaled the need for inpatient intervention, whereas age was inversely associated. For stones ≥ 6 mm, distal location was the only independent predictor favoring discharge, although wide confidence intervals indicate limited precision and should be interpreted critically.

## Supplementary Information

Below is the link to the electronic supplementary material.

Supplementary Material 1

## Data Availability

No datasets were generated or analysed during the current study.

## Declarations

### Generative AI and AI-assisted technologies in the writing process

During the preparation of this work the authors used Grammarly for writing assistance and grammatical correction and ChatGPT for fluency improvement, and statistical assistance with code development, implementation and debugging. All statistical analyses were predefined, conducted, and interpreted by the authors. Following the use of these tools, the authors carefully reviewed and edited all content as necessary and take full responsibility for the final version of the manuscript.
