# Supplementary material for: Determinants of treatment pathway in renal colic: a size-stratified analysis from the emergency department of a tertiary center
Source: Urolithiasis. 2026 Feb 12;54(1):50. doi: 10.1007/s00240-026-01950-1 (PMC12901271; doi:10.1007/s00240-026-01950-1)
Supplement: Supplementary file 1 — Supplementary Material 1 [file 240_2026_1950_MOESM1_ESM.docx]

**Supplementary Material**

| **Variable** | **<6 mm Available (N)** | **<6 mm**  **Missing (%)** | **≥6 mm Available (N)** | **≥6 mm**  **Missing (%)** |
| --- | --- | --- | --- | --- |
| **Age [years]** | 514 | 0.0 | 238 | 0 |
| **Sex** | 513 | 0.2 | 238 | 0 |
| **Arrival at the ED** | 347 | 32.5 | 158 | 33.6 |
| **Fever** | 360 | 30.0 | 157 | 30.0 |
| **Vomiting** | 269 | 47.7 | 102 | 47.7 |
| **CRP [mg/dl]** | 443 | 13.8 | 204 | 13.8 |
| **Leukocytes [G/l]** | 513 | 0.2 | 235 | 0.2 |
| **Creatinine [mg/dl]** | 514 | 0.0 | 235 | 0.0 |
| **eGFR [ml/min]** | 508 | 1.2 | 235 | 1.2 |
| **Urine: Leukocyturia** | 514 | 0.0 | 238 | 0.0 |
| **Location of symptomatic stone** | 506 | 1.6 | 236 | 1.6 |
| **Hydronephrosis** | 514 | 0.0 | 236 | 0.0 |
| **Largest ureteral stone diameter [mm]** | 514 | 0.0 | 238 | 0.0 |

Supplementary Table S1. Data availability and missingness of variables used in univariable and multivariable regression analyses, stratified by stone-size subgroup (<6 mm vs. ≥6 mm).
